# Supplementary figures and images for: Improved Correlation Filter Tracking with Enhanced Features and Adaptive Kalman Filter
Source: Sensors (Basel). 2019 Apr 4;19(7):1625. doi: 10.3390/s19071625 (PMC6479297; doi:10.3390/s19071625)

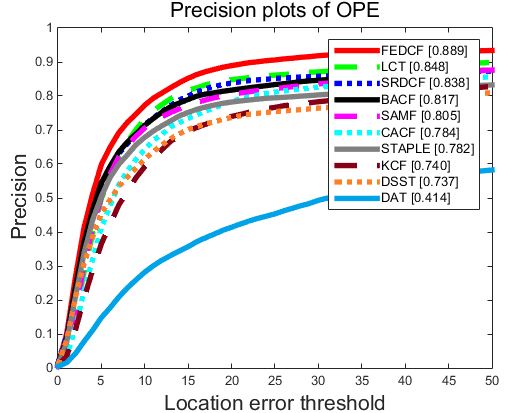

Supplement: Supplementary file 1 [file sensors-19-01625-s001.zip › sensors-456967 4.3/OTB2013/S1.JPG]

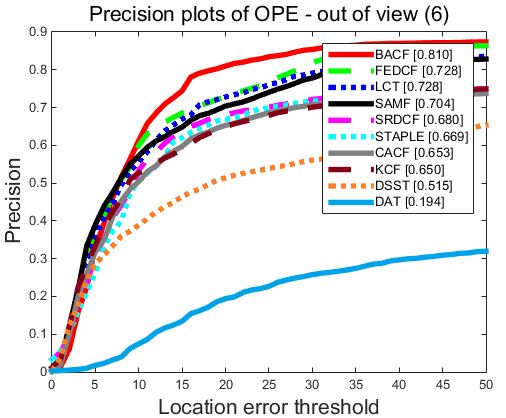

Supplement: Supplementary file 1 [file sensors-19-01625-s001.zip › sensors-456967 4.3/OTB2013/S10.JPG]

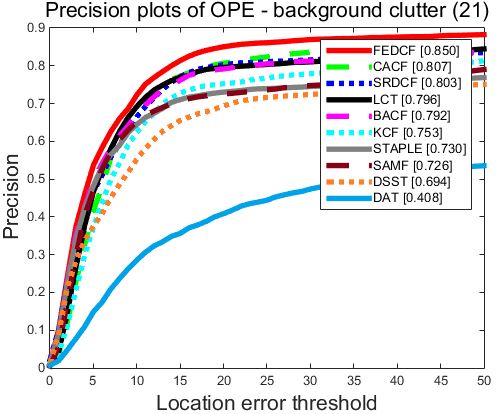

Supplement: Supplementary file 1 [file sensors-19-01625-s001.zip › sensors-456967 4.3/OTB2013/S11.JPG]

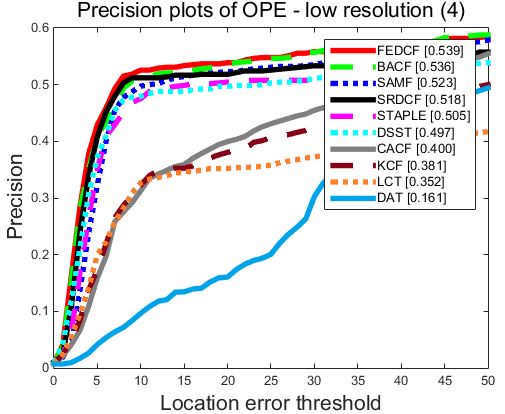

Supplement: Supplementary file 1 [file sensors-19-01625-s001.zip › sensors-456967 4.3/OTB2013/S12.JPG]

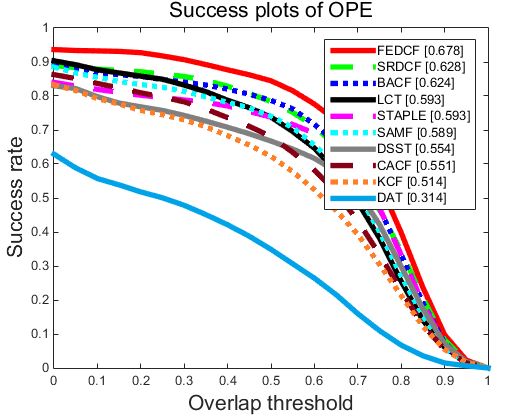

Supplement: Supplementary file 1 [file sensors-19-01625-s001.zip › sensors-456967 4.3/OTB2013/S13.JPG]

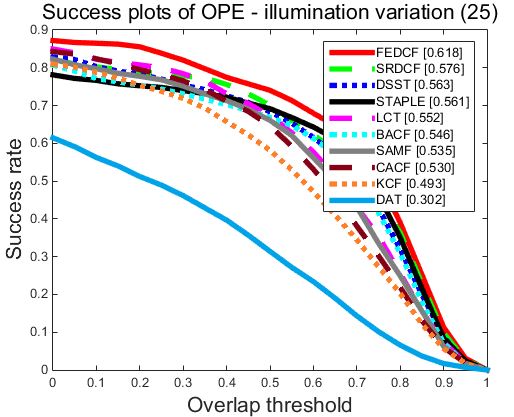

Supplement: Supplementary file 1 [file sensors-19-01625-s001.zip › sensors-456967 4.3/OTB2013/S14.JPG]

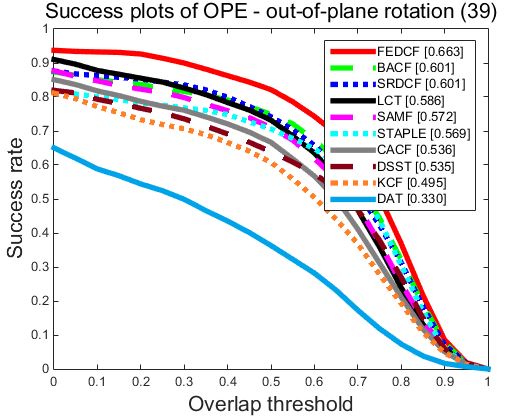

Supplement: Supplementary file 1 [file sensors-19-01625-s001.zip › sensors-456967 4.3/OTB2013/S15.JPG]

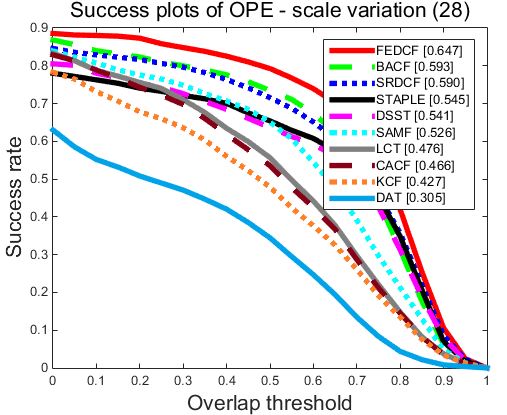

Supplement: Supplementary file 1 [file sensors-19-01625-s001.zip › sensors-456967 4.3/OTB2013/S16.JPG]

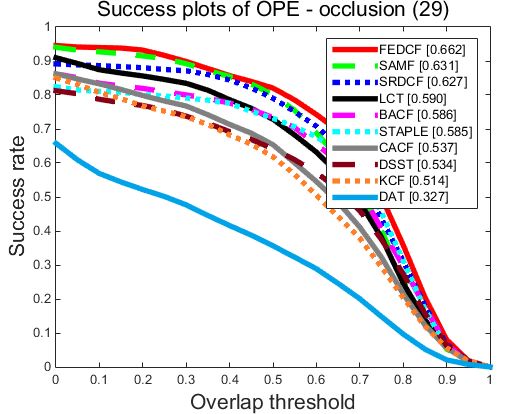

Supplement: Supplementary file 1 [file sensors-19-01625-s001.zip › sensors-456967 4.3/OTB2013/S17.JPG]

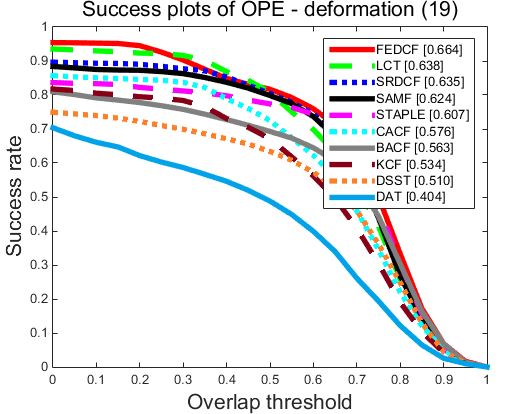

Supplement: Supplementary file 1 [file sensors-19-01625-s001.zip › sensors-456967 4.3/OTB2013/S18.JPG]

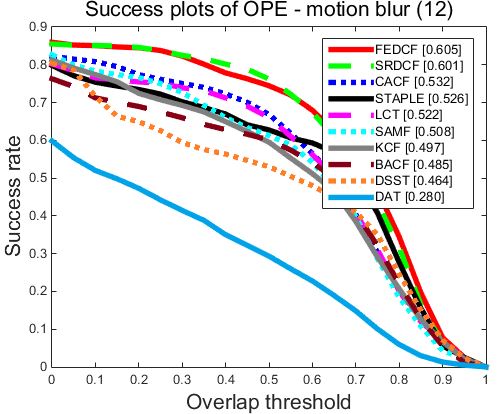

Supplement: Supplementary file 1 [file sensors-19-01625-s001.zip › sensors-456967 4.3/OTB2013/S19.JPG]

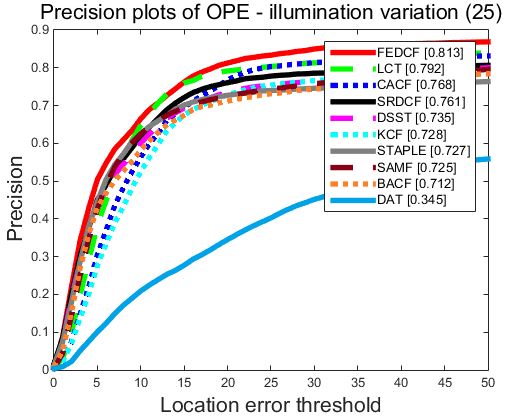

Supplement: Supplementary file 1 [file sensors-19-01625-s001.zip › sensors-456967 4.3/OTB2013/S2.JPG]

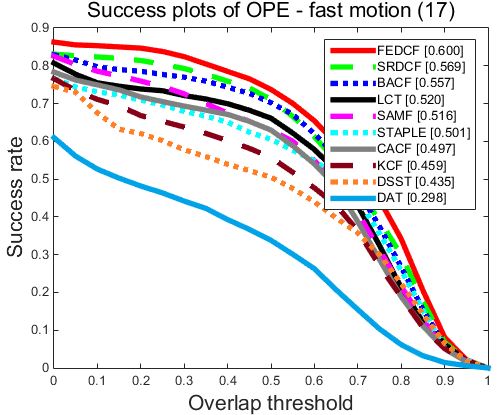

Supplement: Supplementary file 1 [file sensors-19-01625-s001.zip › sensors-456967 4.3/OTB2013/S20.JPG]

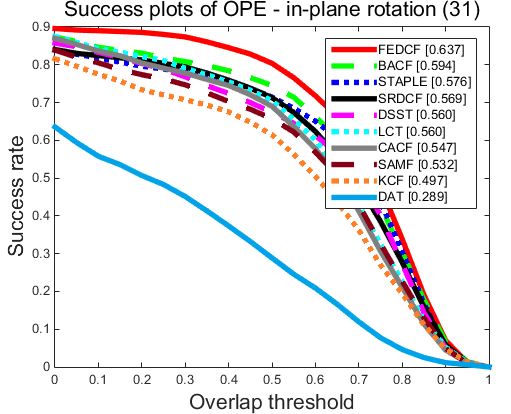

Supplement: Supplementary file 1 [file sensors-19-01625-s001.zip › sensors-456967 4.3/OTB2013/S21.JPG]

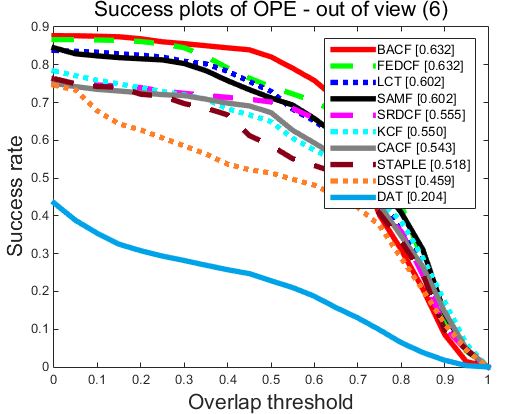

Supplement: Supplementary file 1 [file sensors-19-01625-s001.zip › sensors-456967 4.3/OTB2013/S22.JPG]

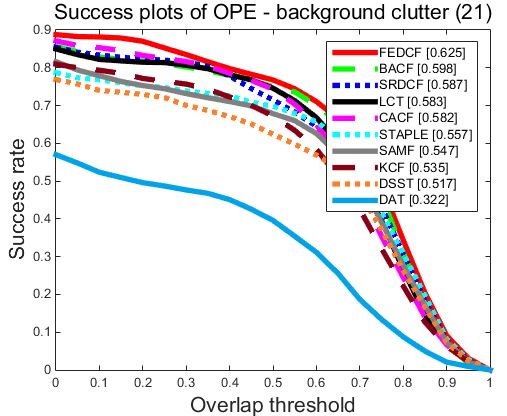

Supplement: Supplementary file 1 [file sensors-19-01625-s001.zip › sensors-456967 4.3/OTB2013/S23.JPG]

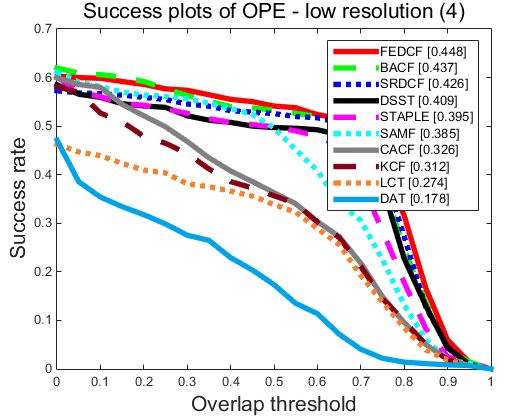

Supplement: Supplementary file 1 [file sensors-19-01625-s001.zip › sensors-456967 4.3/OTB2013/S24.JPG]

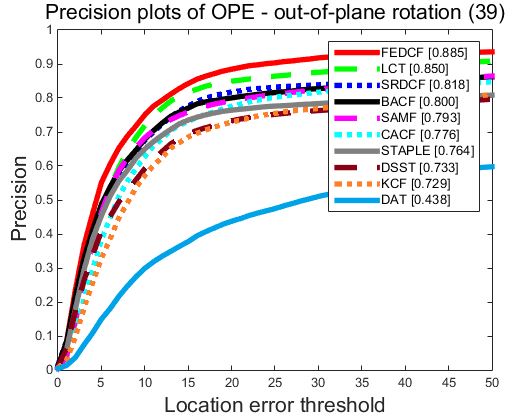

Supplement: Supplementary file 1 [file sensors-19-01625-s001.zip › sensors-456967 4.3/OTB2013/S3.JPG]

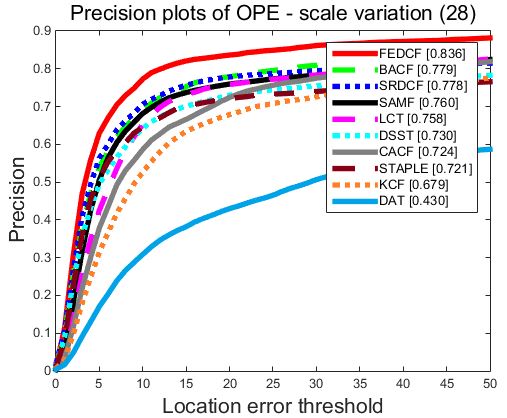

Supplement: Supplementary file 1 [file sensors-19-01625-s001.zip › sensors-456967 4.3/OTB2013/S4.JPG]

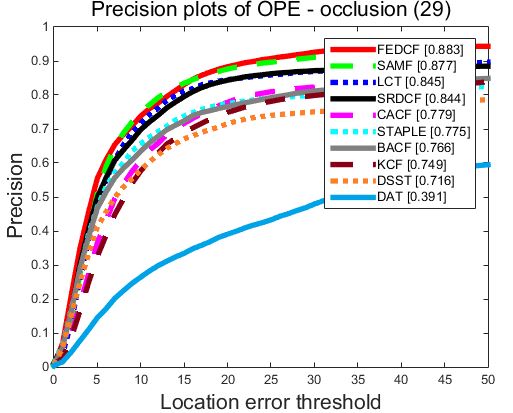

Supplement: Supplementary file 1 [file sensors-19-01625-s001.zip › sensors-456967 4.3/OTB2013/S5.JPG]

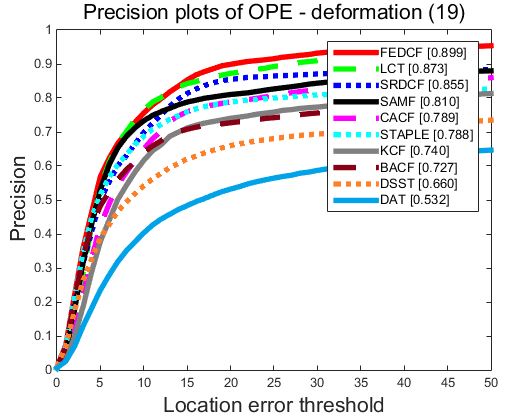

Supplement: Supplementary file 1 [file sensors-19-01625-s001.zip › sensors-456967 4.3/OTB2013/S6.JPG]

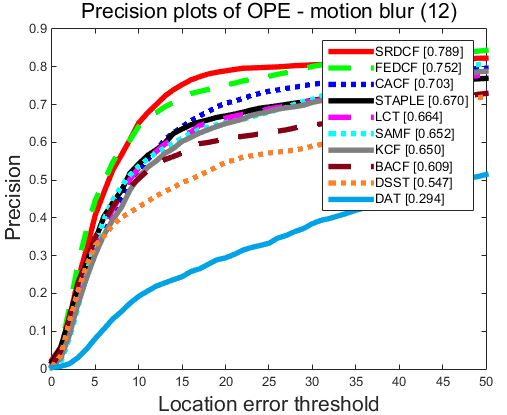

Supplement: Supplementary file 1 [file sensors-19-01625-s001.zip › sensors-456967 4.3/OTB2013/S7.JPG]

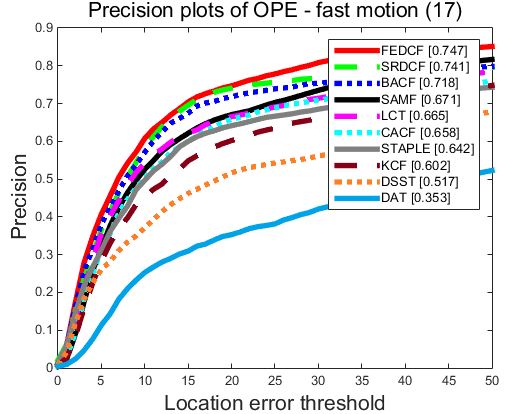

Supplement: Supplementary file 1 [file sensors-19-01625-s001.zip › sensors-456967 4.3/OTB2013/S8.JPG]

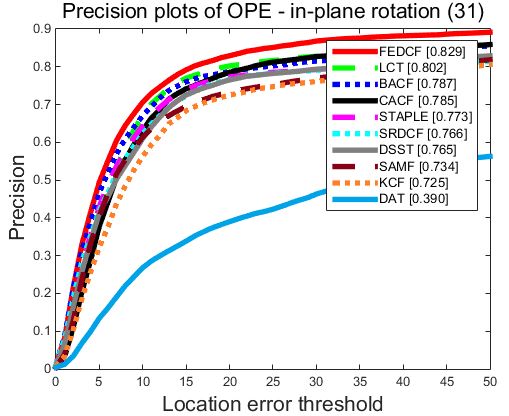

Supplement: Supplementary file 1 [file sensors-19-01625-s001.zip › sensors-456967 4.3/OTB2013/S9.JPG]

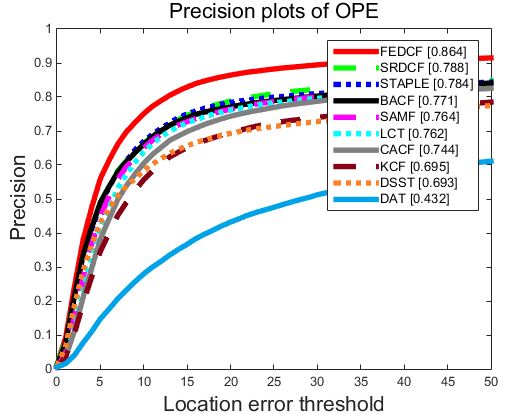

Supplement: Supplementary file 1 [file sensors-19-01625-s001.zip › sensors-456967 4.3/OTB2015/S25.JPG]

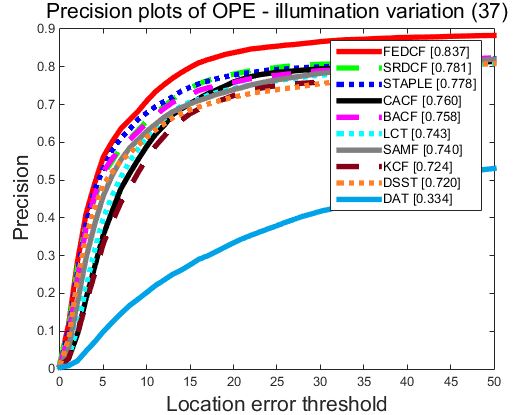

Supplement: Supplementary file 1 [file sensors-19-01625-s001.zip › sensors-456967 4.3/OTB2015/S26.JPG]

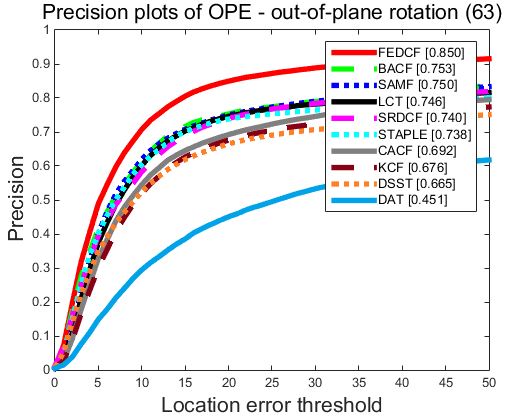

Supplement: Supplementary file 1 [file sensors-19-01625-s001.zip › sensors-456967 4.3/OTB2015/S27.JPG]

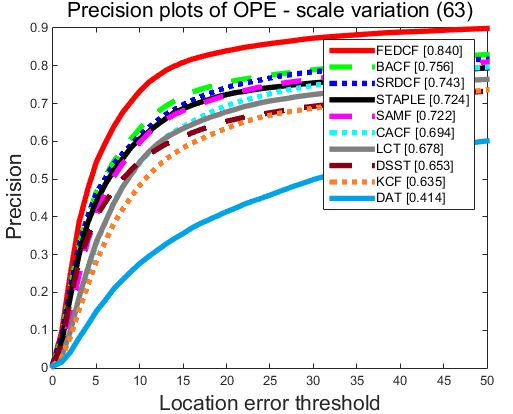

Supplement: Supplementary file 1 [file sensors-19-01625-s001.zip › sensors-456967 4.3/OTB2015/S28.JPG]

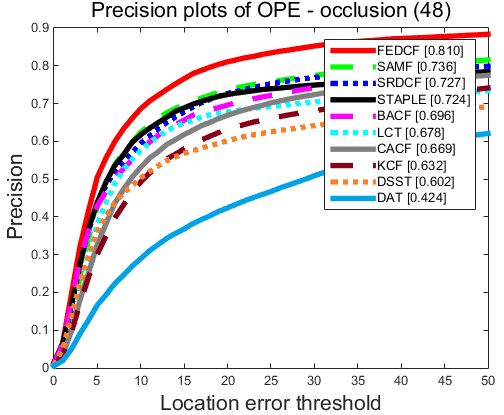

Supplement: Supplementary file 1 [file sensors-19-01625-s001.zip › sensors-456967 4.3/OTB2015/S29.JPG]

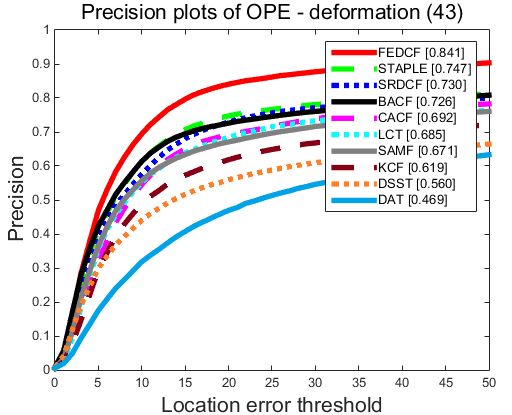

Supplement: Supplementary file 1 [file sensors-19-01625-s001.zip › sensors-456967 4.3/OTB2015/S30.JPG]

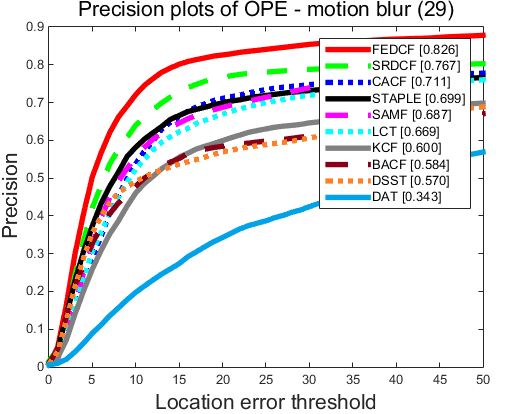

Supplement: Supplementary file 1 [file sensors-19-01625-s001.zip › sensors-456967 4.3/OTB2015/S31.JPG]

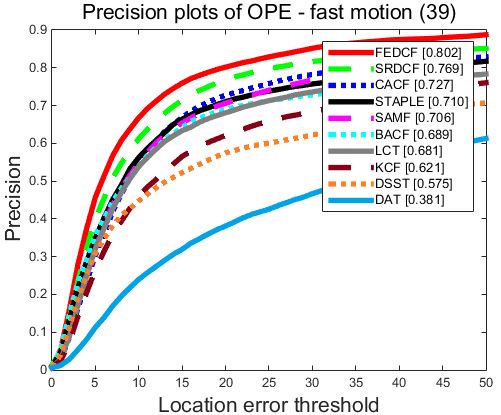

Supplement: Supplementary file 1 [file sensors-19-01625-s001.zip › sensors-456967 4.3/OTB2015/S32.JPG]

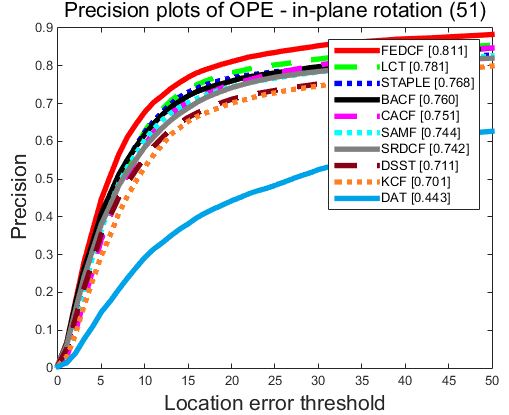

Supplement: Supplementary file 1 [file sensors-19-01625-s001.zip › sensors-456967 4.3/OTB2015/S33.JPG]

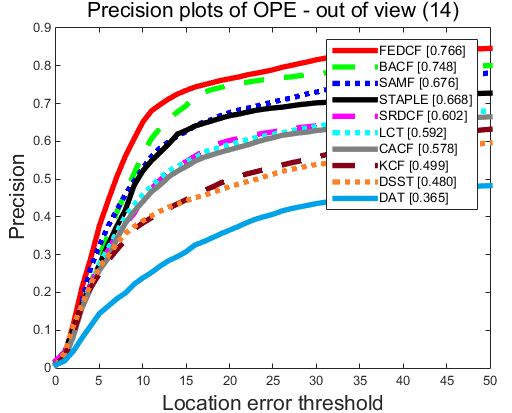

Supplement: Supplementary file 1 [file sensors-19-01625-s001.zip › sensors-456967 4.3/OTB2015/S34.JPG]

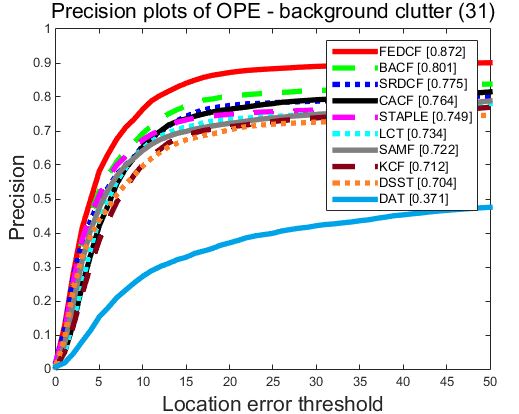

Supplement: Supplementary file 1 [file sensors-19-01625-s001.zip › sensors-456967 4.3/OTB2015/S35.JPG]

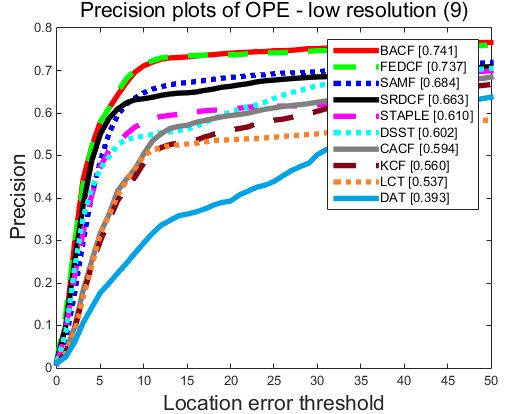

Supplement: Supplementary file 1 [file sensors-19-01625-s001.zip › sensors-456967 4.3/OTB2015/S36.JPG]

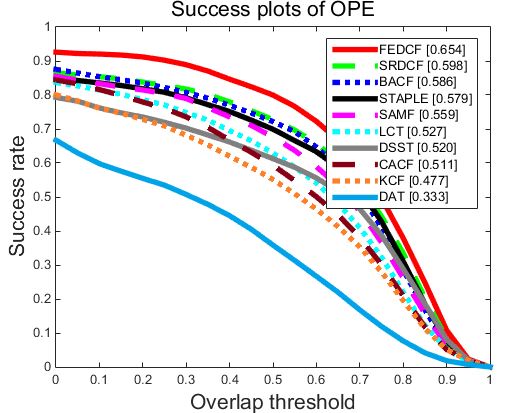

Supplement: Supplementary file 1 [file sensors-19-01625-s001.zip › sensors-456967 4.3/OTB2015/S37.JPG]

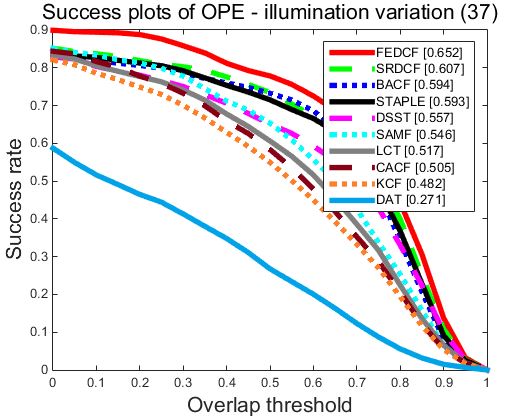

Supplement: Supplementary file 1 [file sensors-19-01625-s001.zip › sensors-456967 4.3/OTB2015/S38.JPG]

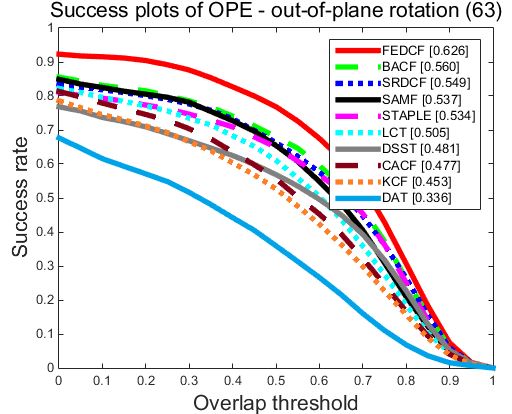

Supplement: Supplementary file 1 [file sensors-19-01625-s001.zip › sensors-456967 4.3/OTB2015/S39.JPG]

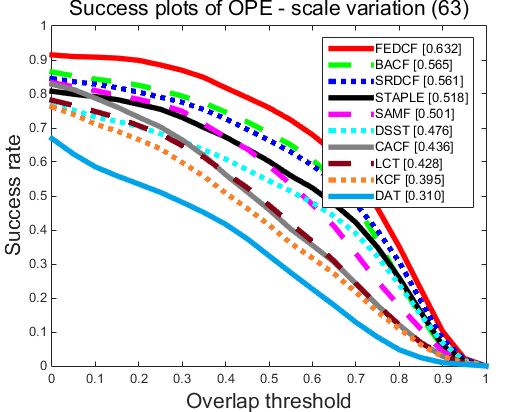

Supplement: Supplementary file 1 [file sensors-19-01625-s001.zip › sensors-456967 4.3/OTB2015/S40.JPG]

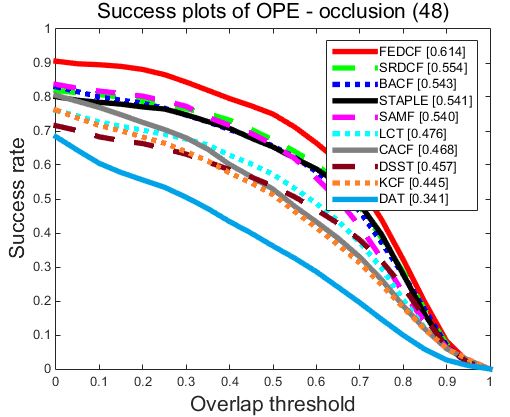

Supplement: Supplementary file 1 [file sensors-19-01625-s001.zip › sensors-456967 4.3/OTB2015/S41.JPG]

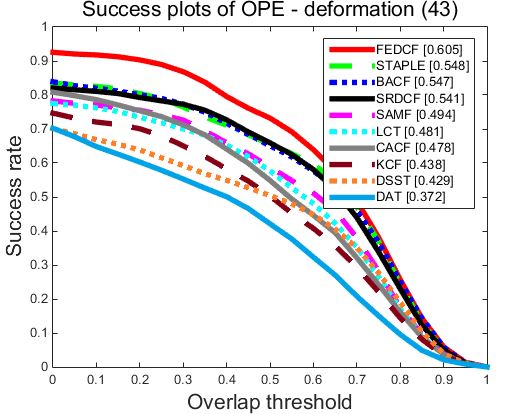

Supplement: Supplementary file 1 [file sensors-19-01625-s001.zip › sensors-456967 4.3/OTB2015/S42.JPG]

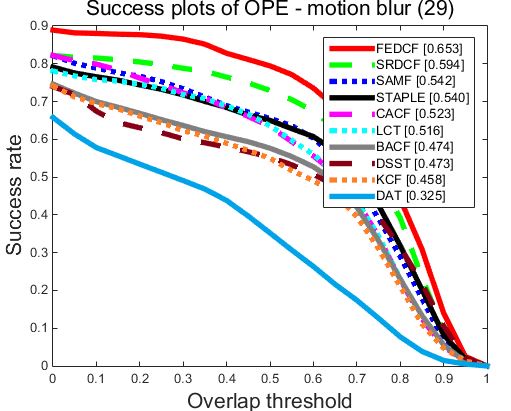

Supplement: Supplementary file 1 [file sensors-19-01625-s001.zip › sensors-456967 4.3/OTB2015/S43.JPG]

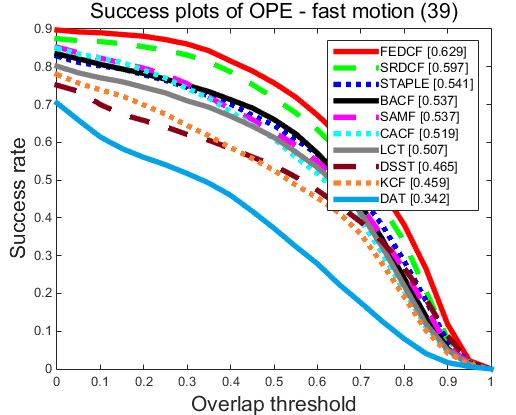

Supplement: Supplementary file 1 [file sensors-19-01625-s001.zip › sensors-456967 4.3/OTB2015/S44.JPG]

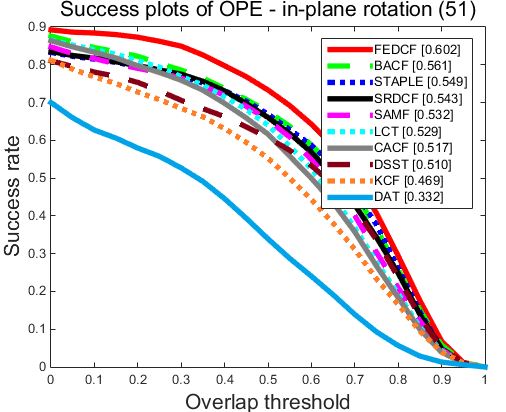

Supplement: Supplementary file 1 [file sensors-19-01625-s001.zip › sensors-456967 4.3/OTB2015/S45.JPG]

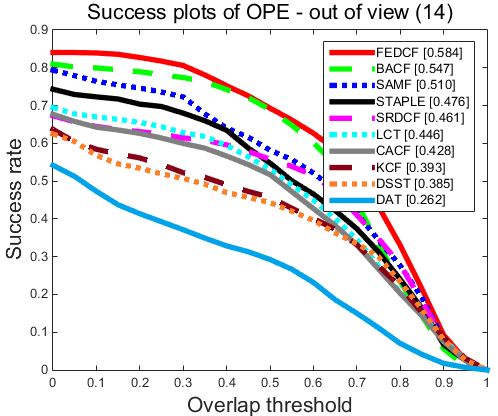

Supplement: Supplementary file 1 [file sensors-19-01625-s001.zip › sensors-456967 4.3/OTB2015/S46.JPG]

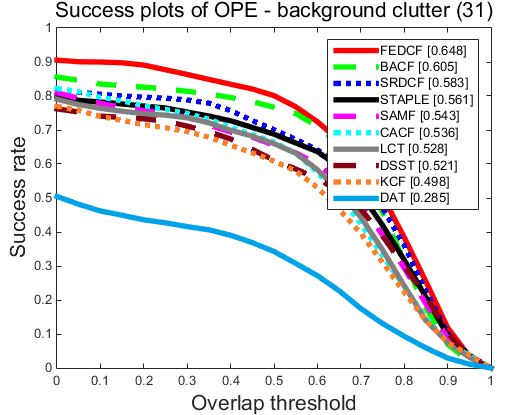

Supplement: Supplementary file 1 [file sensors-19-01625-s001.zip › sensors-456967 4.3/OTB2015/S47.JPG]

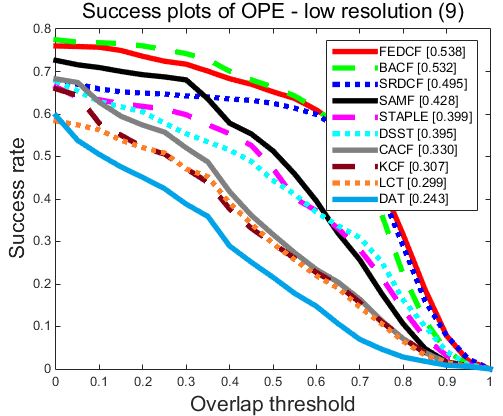

Supplement: Supplementary file 1 [file sensors-19-01625-s001.zip › sensors-456967 4.3/OTB2015/S48.JPG]
